# Supplementary material for: Unraveling the kinetochore nanostructure in Schizosaccharomyces pombe using multi-color SMLM imaging
Source: J Cell Biol. 2023 Jan 27;222(4):e202209096. doi: 10.1083/jcb.202209096 (PMC9930162; doi:10.1083/jcb.202209096)
Supplement: Table S1 — shows S. pombe and E. coli strains used in this study. [file JCB_202209096_TableS1.docx]

| **Strain** | **Genotype** | **Source** |
| --- | --- | --- |
| SP176 (972) | wt h- | gift from Laue lab, Cambridge, UK |
| SP177/ wt h+ | h+, ade6-210, leu1-32, ura4-D18 | (Lando et al., 2012) |
| SP11/ DL70 | h+, ade6-210, leu1-32, ura4-D18, PAmCherry1:cnp1 | (Lando et al., 2012) |
| SP118 | h-, sad1:mScarlet-I:hphMX6 | this study |
| SP145 | h+, leu1-32, ura4-D18, sad1:mScarlet-I:hphMX6, PAmCherry1:cnp1 | this study |
| SP137 | h+, leu1-32, ura4-D18, spc25:mEos3.2-A69T:kanMX6, sad1:mScarlet-I:hphMX6, PAmCherry1:cnp1 | this study |
| SP141 | h+, leu1-32, ura4-D18, mis12:mEos3.2-A69T:kanMX6, sad1:mScarlet-I:hphMX6, PAmCherry1:cnp1 | this study |
| SP144 | h+, leu1-32, ura4-D18, dam1:mEos3.2-A69T:kanMX6, sad1:mScarlet-I:hphMX6, PAmCherry1:cnp1 | this study |
| SP146 | h+, leu1-32, ura4-D18, fta2:mEos3.2-A69T:kanMX6, sad1:mScarlet-I:hphMX6, PAmCherry1:cnp1 | this study |
| SP147 | h+, leu1-32, ura4-D18, fta7:mEos3.2-A69T:kanMX6, sad1:mScarlet-I:hphMX6, PAmCherry1:cnp1 | this study |
| SP150 | h+, leu1-32, ura4-D18, cnp3:mEos3.2-A69T:kanMX6, sad1:mScarlet-I:hphMX6, PAmCherry1:cnp1 | this study |
| SP152 | h+, leu1-32, ura4-D18, ndc80:mEos3.2-A69T:kanMX6, sad1:mScarlet-I:hphMX6, PAmCherry1:cnp1 | this study |
| SP153 | h+, leu1-32, ura4-D18, nnf1:mEos3.2-A69T:kanMX6, sad1:mScarlet-I:hphMX6, PAmCherry1:cnp1 | this study |
| SP154 | h+, leu1-32, ura4-D18, spc7:mEos3.2-A69T:kanMX6, sad1:mScarlet-I:hphMX6, PAmCherry1:cnp1 | this study |
| SP155 | h+, leu1-32, ura4-D18, cnp20:mEos3.2-A69T:kanMX6, sad1:mScarlet-I:hphMX6, PAmCherry1:cnp1 | this study |
| SP109 | h+, ade6-210, leu1-32, ura4-D18, Halo:cnp1 | (Vojnovic, 2016) |
| SP16 | h+, ade6-210, leu1-32, ura4-D18, pREPnmt81-mEos2::Leu2 | (Lando et al., 2012) |
| EC290 | Rosetta DE3 pRSETa mEos3.2-A69T:FtnA | this study |

**Supplementary Table S1: *S. pombe* and *E. coli* strains used in this study**
