# Supplementary material for: Unraveling the kinetochore nanostructure in Schizosaccharomyces pombe using multi-color SMLM imaging
Source: J Cell Biol. 2023 Jan 27;222(4):e202209096. doi: 10.1083/jcb.202209096 (PMC9930162; doi:10.1083/jcb.202209096)
Supplement: Table S2 — list of primers used for constructing the 3C-library and the FtnA protein standard as listed in Table S1. [file JCB_202209096_TableS2.docx]

| **Primer name** | **5’→3’ SEQUENCE + overlap** |
| --- | --- |
| Ade6_F2 | CTCATTAAGCTGAGCTGCCAAG |
| Ade6_R2 | TGCATAGGCGACCATAGACAT |
| AGGSG_mEos3.2_F | gccggaggcagtggttct |
| Cnp3_F1 | GGAAAGATCGAGGTCACAGT |
| Cnp3_F2 | aatgctggtcgctatactgctgtcCAATACTAATAGTGTGTTATGGATTTCG |
| Cnp3_R1_AGGSG | GGGATTTTCCAAACGAACGAgccggaggcagtggt |
| Cnp3_R2 | AAAGTCAAATCTAACGGTCGC |
| Cnp20_F1 | TTCAACACTTGTGCTACCGGAAA |
| Cnp20_F2 | aatgctggtcgctatactgctgtcTGCGTACTTCTCCTTTACATTCATC |
| Cnp20_R1_AGGSG | ACCTCCGGCAATTAAGAGAACCgccggaggcagtggt |
| Cnp20_R2 | CGCTTGATTCGATACACTTACAAGT |
| Dam1-GFP F1 | TGCCGAAAGCGCTGTAGAA |
| Dam1F2 | aatgctggtcgctatactgctgtcATTTATTTAAGCAAGGGAGACTGGTTG |
| Dam1_R1_Overhang_AGGSG_D2 | AGAAACCTATTCCGCTTCCAGAgccggaggcagtggttct |
| Dam1R2 | TAGCTTCTCCAATCTTCAATTTCCA |
| fta2_F1 | CATGGACGCTCAATGTTTCT |
| fta2_F2 | aatgctggtcgctatactgctgtcGAAGGATAAATTGATATTTTTAACATGGTT |
| fta2_R1_AGGSG | GGCATTATTTAAACCTCATTTGGGGgccggaggcagtggt |
| fta2_R2 | AGTTCTTTTGGCAGAATGGG |
| Fta7_F1 | TTCAGACTCCAACGATTTCTC |
| Fta7_F2 | aatgctggtcgctatactgctgtcACATAGAAAAGCTAGAGCTTAAGAC |
| Fta7_R1_AGGSG | AGTCATCCAAACTTAAGATAAAGAATATCgccggaggcagtggt |
| Fta7_R2 | GAGTTTAGGGTAGGGTAAGCA |
| KanR_cassette_R | Aatgctggtcgctatactgctgtc |
| Mis12_F1 | TCTGCAGCATGCCGTTAAAAG |
| Mis12_F2 | aatgctggtcgctatactgctgtcTACTAATCAACTAGCTAAAGTCTTGAGATG |
| Mis12_R1_AGGSG | CGGACATACTGACGAGCCTgccggaggcagtggt |
| Mis12_R2 | TCTGACCCATTAACTCCAAATCTGT |
| Ndc80_F1 | ACAACAGCTCAAACTTTCTTCG |
| Ndc80_F2 | aatgctggtcgctatactgctgtcATTCTATTCATCGTATTGTGCTGTC |
| Ndc80_R1_AGGSG | ACCTATCTCGTTCGGAACTGgccggaggcagtggt |
| Ndc80_R2 | ACGAACTGTTTTGGCTAAAAATTTG |
| Nnf1_F1 | AAGCTTAATCAGGATCTGTTGG |
| Nnf1_F2 | aatgctggtcgctatactgctgtcAAGAAGTAAATTTCTAATCAGTTGCA |
| Nnf1_R1_AGGSG | AACGAACAAGGAAATATAGAACGTgccggaggcagtggt |
| Nnf1_R2 | CTCAAAAACATTCCAACGCAA |
| Spc7_F1 | TTGAGCTATACCTGCGTTCGG |
| Spc7_F2 | aatgctggtcgctatactgctgtcATATTAATGGGAATGATTAGCTATGCTGC |
| Spc7_R1_AGGSG | CTTGTCTTACTGTTTGGAACAAATACAGCgccggaggcagtggt |
| Spc7_R2 | AAACCCGTAATGCGCTACAAAA |
| Spc25_F1 | ATCAATCTTGCTGAAAGGGATTA |
| Spc25_F2 | aatgctggtcgctatactgctgtcAGATCTTCTTCTTGTTTAACATAAACTT |
| Spc25_R1_AGGSG | TAGAAAGGATCTGTCTCAATTGATTgccggaggcagtggt |
| Spc25_R1_D2 | TAGAAAGGATCTGTCTCAATTGATTgccggaggcagtggtATG |
| Spc25_R2 | AACTGGTGGAATCCATGGT |
| mEos3.2_F_pRSET | ACGATAAGGATCGATGGGGATCCATGtctgccattaaaccgga |
| mEos3.2_R_FtnA | ggatgctccgctagccttgcgacgcgcattatcc |
| pRSET_F_preFtnA_68 | aaggctagcggagcatcc |
| pRSET_R_blunt_68 | GGATCCCCATCGATCCTTATCGT |

**Supplementary Table S2: List of primers used for constructing the 3C-library and the FtnA protein standard as listed in table S1**
