# Supplementary material for: Unraveling the kinetochore nanostructure in Schizosaccharomyces pombe using multi-color SMLM imaging
Source: J Cell Biol. 2023 Jan 27;222(4):e202209096. doi: 10.1083/jcb.202209096 (PMC9930162; doi:10.1083/jcb.202209096)
Supplement: Table S3 — shows weighted mean of localization counts for POIs belonging to the same KT subcomplex. [file JCB_202209096_TableS3.docx]

| **complex** | **components** | **expected ratios** | **measured ratios** | **weighted mean** | **STD** |
| --- | --- | --- | --- | --- | --- |
| **COMAc** | fta2, fta7, mal2, mis17 | 1:1:1:1 | 1:1.0:NA:NA | 56.0 | 28.2 |
| **MINDc** | mis12, nnf1, mis13, mis14 | 1:1:1:1 | 1:1.3:NA:NA | 85.1 | 43.1 |
| **NDC80c** | ndc80, nuf2, spc24, spc25 | 1:1:1:1 | 1:NA:NA:1.1 | 111.2 | 51.7 |

**Supplementary Table S3: Weighted mean of localization counts for POIs belonging to the same kinetochore subcomplex**
