# Supplementary material for: Unraveling the kinetochore nanostructure in Schizosaccharomyces pombe using multi-color SMLM imaging
Source: J Cell Biol. 2023 Jan 27;222(4):e202209096. doi: 10.1083/jcb.202209096 (PMC9930162; doi:10.1083/jcb.202209096)
Supplement: Table S4 — shows POI copy number ratios between different KT subcomplexes. [file JCB_202209096_TableS4.docx]

| **complexes** | **ratio** | **reference** | **host** | **method** | **cell cycle stage** |
| --- | --- | --- | --- | --- | --- |
| **cnp20^CENP-T^ : COMA** | 1 : 0.9 | this study | *S. pombe* | SMLM imaging | Meta- to Anaphase A |
|  | 1: 2.0 | (Cieslinski et al., 2021) | *S. cerevisiae* | SMLM imaging | Metaphase |
| **MIND : spc7^KNL1^** | 1 : 1.1 | this study | *S. pombe* | SMLM imaging | Meta- to Anaphase A |
|  | 1 : 0.7 | (Joglekar et al., 2008) | *S. pombe* | fluorescence ratio | G2 to Metaphase |
|  | 1 : 1.0 | (Joglekar et al., 2008) | *S. pombe* | fluorescence ratio | Anaphase to Telophase |
|  | 1 : 0.7 | (Lawrimore et al., 2011) | *S. pombe* | corrected from Joglekar et al. 2008 | G2 to Metaphase |
|  | 1 : 1.3 | (Joglekar et al., 2006) | *S. cerevisiae* | fluorescence ratio | Metaphase |
|  | 1 : 1.1 | (Joglekar et al., 2006) | *S. cerevisiae* | fluorescence ratio | Anaphase |
|  | 1 : 1.0 | (Lawrimore et al., 2011) | *S. cerevisiae* | fluorescence ratio | Anaphase |
|  | 1 : 0.7 | (Dhatchinamoorthy et al., 2017) | *S. cerevisiae* | fluorescence ratio | Anaphase |
|  | 1 : 1.1 | (Cieslinski et al., 2021) | *S. cerevisiae* | SMLM imaging | Metaphase |
|  | 1 : 0.8 | (Johnston et al., 2010) | *Chicken DT40* | fluorescence ratio | Metaphase |
|  | 1 : 0.8 | (Lawrimore et al., 2011) | *Chicken DT40* | fluorescence ratio | Metaphase |
|  | 1 : 0.8 | (Emanuele et al., 2005) | *X. laevis* | Biochemical assay | unsynchronized |
| **COMA : MIND** | 1 : 1.5 | this study | *S. pombe* | SMLM imaging | Meta- to Anaphase A |
|  | 1 : 1.6 | (Joglekar et al., 2008) | *S. pombe* | fluorescence ratio | G2 to Metaphase |
|  | 1 : 0.8 | (Joglekar et al., 2008) | *S. pombe* | fluorescence ratio | Anaphase to Telophase |
|  | 1 : 1.6 | (Lawrimore et al., 2011) | *S. pombe* | corrected from Joglekar et al. 2008 | G2 to Metaphase |
|  | 1 : 2.2 | (Joglekar et al., 2006) | *S. cerevisiae* | fluorescence ratio | Metaphase |
|  | 1 : 2.3 | (Joglekar et al., 2006) | *S. cerevisiae* | fluorescence ratio | Anaphase |
|  | 1 : 2.3 | (Lawrimore et al., 2011) | *S. cerevisiae* | fluorescence ratio | Anaphase |
|  | 1 : 2.3 | (Dhatchinamoorthy et al., 2017) | *S. cerevisiae* | fluorescence ratio | Anaphase |
|  | 1 : 1.8 | (Cieslinski et al., 2021) | *S. cerevisiae* | SMLM imaging | Metaphase |

**Supplementary Table S4: POI copy number ratios between different kinetochore subcomplexes**
