# Supplementary material for: Unraveling the kinetochore nanostructure in Schizosaccharomyces pombe using multi-color SMLM imaging
Source: J Cell Biol. 2023 Jan 27;222(4):e202209096. doi: 10.1083/jcb.202209096 (PMC9930162; doi:10.1083/jcb.202209096)
Supplement: Table S5 — shows comparison of protein cluster distances between our study in S. pombe and the study of Cieslinski et al. (2023) in S. cerevisiae. [file JCB_202209096_TableS5.docx]

|  | **Kinetochore subcomplex** | **POI**  (homolog in  *S. cerevisiae*) | **distance [nm] ± STD**  to *S.pombe* C-term spc7^KNL1^ (spc105)*,* this study | **distance [nm] ± SEM**  to *S. cerevisiae* C-term spc7^KNL1^ (spc105), Cieslinski et al. |
| --- | --- | --- | --- | --- |
| **cnp1^CENP-A^** |  | **cnp1^CENP-A^ (cse4)** | N-term: -25.9 ± 1.2 | C-term: -16.9 ± 1.3 |
| **CCAN** | **CBF3**  (only in cerevisiae) | **N/A (cep3)** | / | -21.1 ± 1.7 |
|  |  | **cnp3 (mif2)** | / | -23.8 ± 2.0 |
|  | **CENP-T/cnn1** | **cnp20^CENP-T^ (cnn1)** | -17.6 ± 2.7 | -20.1 ± 2.7 |
|  | **COMA** | **fta2^CENP-P^ (ctf19)** | -14.5 ± 2.2 | -14.9 ± 1.7 |
|  |  | **fta7^CENP-Q^ (okp1)** | -14.2 ± 3.1 | -13.4 ± 1.4 |
|  | **CENP-N/Chl4** | **mis15 (chl4)** | / | -23.5 ± 2.9 |
| **KMN** | **KNL1/Spc105** | **spc7^KNL1^ (spc105)** | 0 | 0 |
|  | **MIND** | **nnf1^PMF1^** | 1.2 ± 1.8 | 4.8 ± 2.6 |
|  |  | **mis12 (mtw1)** | 2.1 ± 2 | 4.3 ± 0.6 |
|  |  | **mis13 (dsn1)** | / | 3.1 ± 0.6 |
|  |  | **mis14 (nsl1)** | / | 6.5 ± 1.5 |
|  | **NDC80** | **spc25** | -4 ± 2.5 | -2.5 ± 0.8 |
|  |  | **ndc80^HEC1^** | 10.3 ± 1.9 | 13.6 ± 1.2 |
|  |  | **nuf2** | / | 16.9 ± 1.5 |
| **Dam1/DASH** | **Dam1/DASH** | **dam1** | 38.3 ± 1.8 | / |
|  |  | **ask1** | / | 44.3 ± 1.8 |

**Supplementary Table S5:** Comparison of protein cluster distances between our study in *S. pombe* and the study of Cieslinski et al. in *S. cerevisiae*. The studies used different reference proteins. Whereas our studies used N-terminal cnp1^CENP-A^ as the reference, Cieslinski et al used C-terminal spc7^KNL1^. In the upper table, we thus converted our numbers and errors into the Cieslinski et al. reference frame by using our distance between C-terminal spc7^KNL1^ and N-terminal cnp1^CENP-A^. From the literature, it is known that the intramolecular C- to N-terminal cnp1^CENP-A^ distance is about 3 - 5 nm (Migl et al., 2020; Sekulic et al., 2010; Tachiwana et al., 2011; Yan et al., 2019) which should be considered when comparing the numbers in the above table.
